# Observation and simulation of atmospheric gravity waves exciting subsequent tsunami along the coastline of Japan after Tonga explosion event

**DOI:** 10.1038/s41598-022-25854-3

**Published:** 2022-12-26

**Authors:** Yasuhiro Nishikawa, Masa-yuki Yamamoto, Kensuke Nakajima, Islam Hamama, Hiroaki Saito, Yoshihiro Kakinami, Masumi Yamada, Tung-Cheng Ho

**Affiliations:** 1 School of Systems Engineering, Kochi University of Technology, Kami, Kochi Japan; 2 Faculty of Sciences, Kyushu University, Fukuoka, Japan; 3 Egyptian National Data Center, National Research Institute of Astronomy and Geophysics, Helwan, Cairo Egypt; 4 Department of Cosmosciences, Hokkaido University, Sapporo, Hokkaido Japan; 5 Space Information Center, Hokkaido Information University, Ebetsu, Hokkaido Japan; 6 Disaster Prevention Research Institute, Kyoto University, Uji, Japan

**Keywords:** Natural hazards, Atmospheric dynamics

## Abstract

Tsunamis are commonly generated by earthquakes beneath the ocean floor, volcanic eruptions, and landslides. The tsunami following the Tonga eruption of 2022 is believed to have been excited by atmospheric pressure fluctuations generated by the explosion of the volcano. The first, fast-traveling tsunami was excited by Lamb waves; however, it has not been clarified observationally or theoretically which type of atmospheric fluctuations excited more prominent tsunami which followd. In this study, we investigate atmospheric gravity waves that possibly excited the aforementioned subsequent tsunami based on observations and atmosphere-ocean coupling simulations. The atmospheric fluctuations are classified as Lamb waves, acoustic waves, or gravity waves. The arrival time of the gravity wave and the simulation shows that the gravity wave propagated at a phase speed of 215 m/s, coinciding with the tsunami velocity in the Pacific Ocean, and suggesting that the gravity wave resonantly excited the tsunami (Proudman resonance). These observations and theoretical calculations provide an essential basis for investigations of volcano-induced meteotsunamis, including the Tonga event.

## Introduction

Inaudible sound with a frequency of less than 20 Hz is known as infrasound, and is generated by large-scale natural phenomena such as earthquakes, tsunamis, and volcanic eruptions. Infrasound is used for remote sensing of natural disasters because it can travel far with little attenuation. Generally, infrasound is excited when a large tsunami occurs, but in the case of the Tonga eruption, the opposite happened. At around 4:15 (UTC) on January 15, 2022, the volcano Hunga Tonga-Hunga Ha’apai in Tonga erupted, completely destroying the volcanic island. At approximately 11:00 (UTC), 7 h after the eruption, sea-level changes began on the coastline of Japan, and, subsequently, at around 1500UT, their amplitudes increased to 0.9 m and 1.2 m in at least two local ports (Kuji 36.5$^{\circ }$ N 140.6$^{\circ }$ E and Amami 28.3$^{\circ }$ N 129.4$^{\circ }$ E). Local topographic effects, such as reflections in bays, can cause larger tsunamis^1,2^, but that theory alone does not fully explain the excitation of offshore tsunamis. The first tsunami that was observed in Japan had a small amplitude and is considered to have been excited by Lamb waves^3,4^, but the excitation mechanism for the subsequent tsunami with a larger amplitude is still an open question.

**Figure 1:**
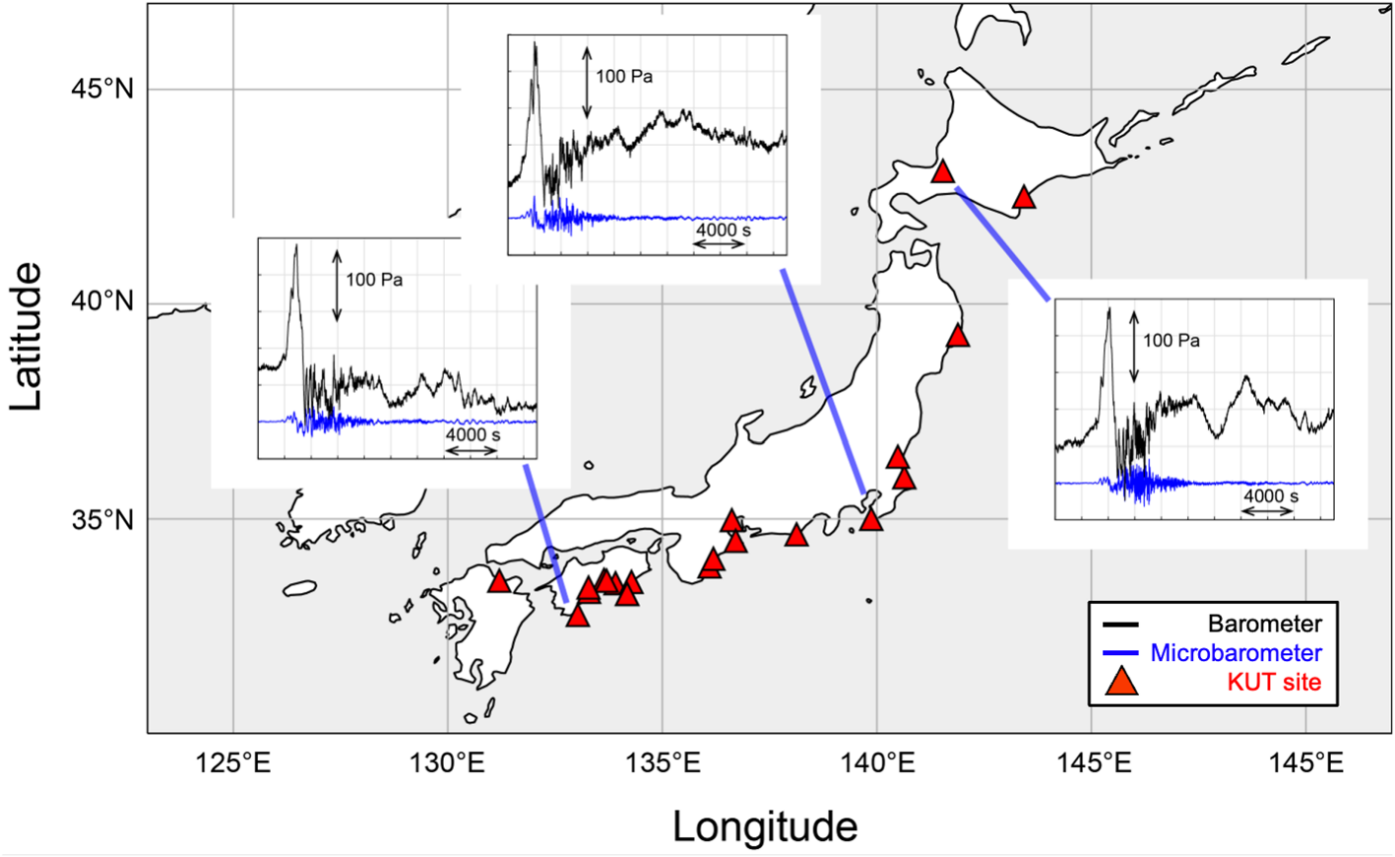
Location of Kochi University of Technology (KUT) infrasound sensors and their observed data after the Tonga volcanic eruption. The KUT installed more than 30 infrasound sensors to form a Japan-wide infrasound observation network. Every site has a SAYA INF01-type comprehensive sensor that contains a membrane-type infrasound sensor, a barometer, a thermometer, and a three-component accelerometer of small MEMS sensor chips. These observation sites are 7700 to 8400 km away from Tonga volcano, and the pressure fluctuations were monitored approximately 7 hours after the eruption. Examples of time series of pressure perturbation are shown; the observed signals had similar waveforms regardless of their locations. The map is created with Matlab R2022B (www.mathworks.com).

The impulsive shock wave, directly resulting from the volcanic eruption, turned into long-period atmospheric waves. Their pressure fluctuations were observed worldwide as a spreading ripple. Some of these long-period atmospheric waves, trapped within the lower  100 km of the atmosphere, are known to propagate over long distances with little attenuation. These long-period waves propagated over the Pacific Ocean and reached Japan, travelling approximately 8,000 km from the volcano. Twenty-five comprehensive infrasound sensors (SAYA INF01) installed along the Pacific coastline of Japan by Kochi University of Technology (KUT)^5^ detected these pressure waves (Fig. 1). Long-period atmospheric waves including infrasound and atmospheric gravity waves are generated by geophysical events, such as volcanic eruptions^6,7^, thunder tsunamis^8,9^, earthquakes^10^, landslides, meteoroid impacts^11,12^, and artificial sources including rocket launches and chemical explosions^13^. Here, we refer to infrasound as sound waves with a frequency of less than 20 Hz, the lower limit of audible sound, which propagate at the speed of sound through the atmosphere as pressure changes.

**Figure 2:**
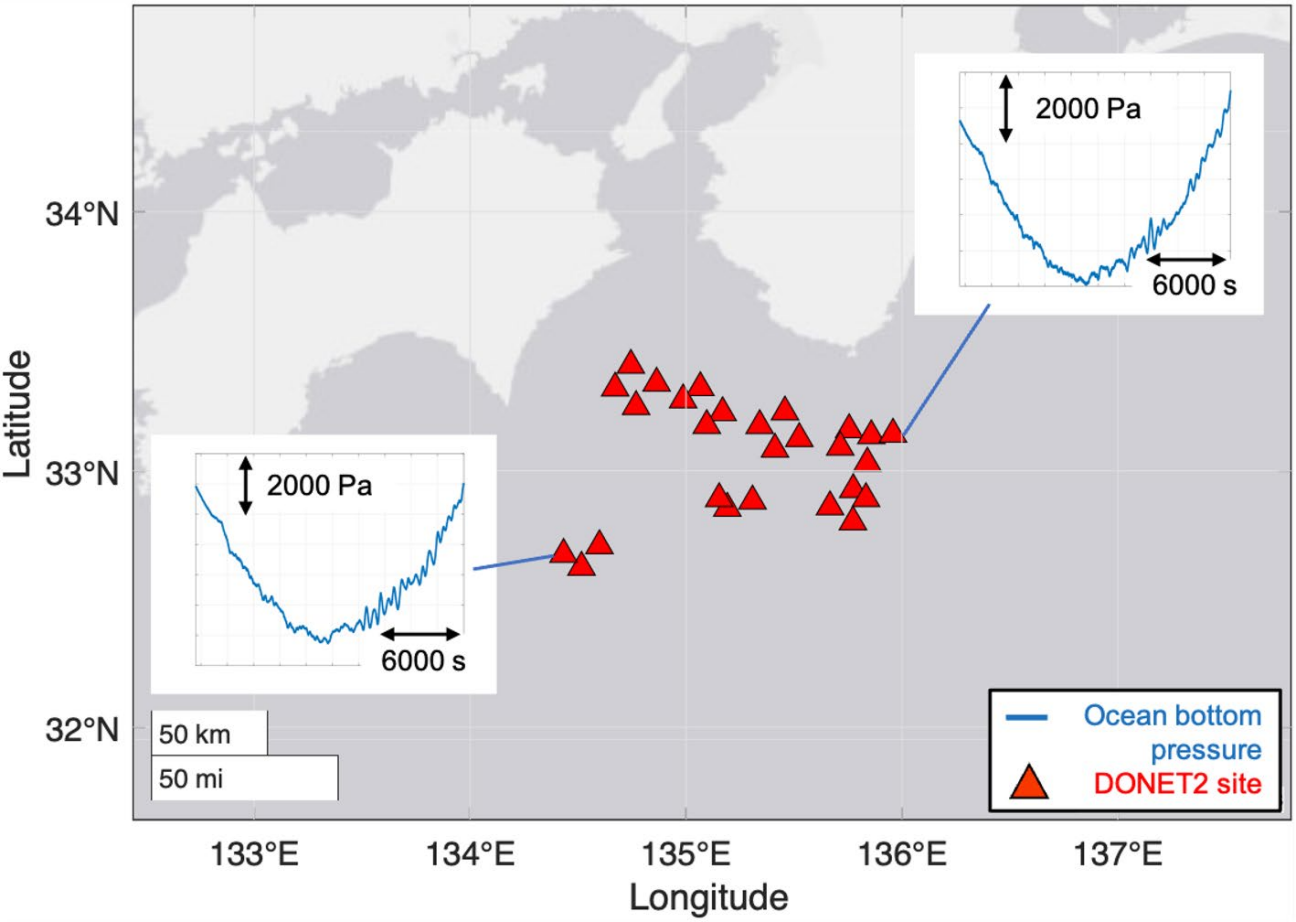
Location of DONET2 (Dense Oceanfloor Network system for Earthquakes and Tsunamis) sites. These 27 ocean bottom pressure gauges are installed for the early detection of tsunamis by observing the vertical movement of the sea surface through the observation of changes in water pressure. The map is created with Matlab R2022B (www.mathworks.com).

In the following, we discuss the whole event from the explosive eruption to the development of the observed tsunami based on coupled atmospheric and oceanic waves, based on both observations and numerical modelling. We also examine the ocean-bottom pressure fluctuations observed by DONET’s (Dense Oceanfloor Network System for Earthquakes and Tsunamis) seafloor pressure gauges (Fig. 2). More specifically, we perform a frequency analysis of the observed infrasound/gravity wave datasets to identify long-period Lamb waves and gravity waves, and short-period acoustic waves with different propagation paths to determine the characteristics of each type of wave, mainly in terms of velocity differences and paths. We also perform a numerical simulation to understand the eruption of Tonga and the atmosphere-induced meteorological tsunami.

## Methods

### Atmosphere-tsunami coupling model

Here we describe the numerical experiment presented in “Gravity waves excited Tsunami over the Pacific Ocean” section.

#### Basic equations of atmosphere

We consider the two-dimensional motion of air under gravity forced by the heating to represent the effect of a volcanic eruption. Equations of motion are

1 $$\begin{aligned}{} & {} \frac{\partial u}{\partial t}+u\frac{\partial u}{\partial x}+w\frac{\partial u}{\partial z} = -\frac{\partial 1}{\partial \rho }\frac{\partial p}{\partial x'}, \end{aligned}$$

2 $$\begin{aligned}{} & {} \frac{\partial w}{\partial t}+u\frac{\partial w}{\partial x}+w\frac{\partial w}{\partial z} = -\frac{\partial 1}{\partial \rho }\frac{\partial p}{\partial z}-g- \varepsilon {w}, \end{aligned}$$

where *t* is time, *x* is horizontal coordinate, *z* is vertical coordinate measured upward from the lower boundary, *u* and *w* are horizontal and vertical components of velocity, respectively, *p* is pressure, $\rho$ is density, *g*=9.8 $\text {m}\,\text {s}^{-2}$ is an acceleration of gravity. The last term on the right-hand side of Eq. (1) is the damping term to prevent the reflection of waves from the top boundary described later. The equation of continuity is

3 $$\begin{aligned} \frac{\partial \rho }{\partial t}=- \frac{\partial (\rho u)}{\partial x}-\frac{\partial (\rho w)}{\partial z}, \end{aligned}$$

and, the thermodynamic equation is

4 $$\begin{aligned} \frac{\partial \theta }{\partial t}+u \frac{\partial \theta }{\partial x}+w \frac{\partial \theta }{\partial z}= \frac{1}{c_p\rho \Pi }Q, \end{aligned}$$

where *Q* is heating per unit volume, $c_p$=1004 $\text {J}$ $\text {K}^{-1}\text {kg}^{-1}$ is specific heat per unit mass air under constant pressure, $\Pi$ and $\theta$ are a non-dimensional pressure (so-called Exner function) and potential temperature, respectively, which are defined as

5 $$\begin{aligned} \Pi =\Bigg ({\frac{p}{P}}\Bigg )^{\frac{R}{c_p}}, \end{aligned}$$

and

6 $$\begin{aligned} \theta =\frac{T}{\Pi }, \end{aligned}$$

with the help of the equation of state of the ideal gas, which is

7 $$\begin{aligned} p=\rho RT, \end{aligned}$$

where $R=286$ $\text {J} \text {K}^{-1}\text {kg}^{-1}$ is gas constant per unit mass air and $P=10^{5} Pa$ is a reference pressure.

We define a motionless basic state, whose values of corresponding variables depend only on *z* and are noted by subscript zero. We specify it by vertical distribution of temperature $T_0(z)$ and the pressure at the lower boundary $p_0(0)$ and calculate by the help of hydrostatic equation

8 $$\begin{aligned} 0=\frac{RT_0(z)}{p_{0}(z)}\frac{dp_0(z)}{dz}-g. \end{aligned}$$

Then, the equations for small amplitude perturbation around the basic state, which are noted by prime, can be written as

9 $$\begin{aligned}{} & {} \frac{\partial u^*}{\partial t}=\frac{\partial p'}{\partial x'}, \end{aligned}$$

10 $$\begin{aligned}{} & {} \frac{\partial w^*}{\partial t}=-\frac{\partial \rho '^*}{\partial z}-gp -\varepsilon {w^*}, \end{aligned}$$

11 $$\begin{aligned}{} & {} \frac{\partial \rho '}{\partial t}=-\frac{\partial u^*}{\partial x}-\frac{\partial w^*}{\partial z}, \end{aligned}$$

12 $$\begin{aligned}{} & {} \frac{\partial \theta *}{\partial t}=- \frac{N^2}{g}w^*+\frac{Q}{c_{p}T_{0}(z)'}, \end{aligned}$$

13 $$\begin{aligned}{} & {} p'={c_s}^2(\rho '+\theta *), \end{aligned}$$

where $u^* =\rho _{0}u'$,$w^*=\rho _{0}w'$,$\theta ^*=\rho _{0}\theta '/\theta _0$, and, $N^2=(g/\theta _{0}) d\theta /dz$ and $c_s^2=(c_p/c_v)RT$ are the square of buoyancy frequency and sound velocity, respectively, with $c_v$ being specific heat per unit mass air under constant volume.

#### Coupling of atmosphere and tsunamis

In order to integrate the equations above in time, we require the vertical boundary conditions of $w^*$. At the top boundary $z=z_{top}$, we specify $w^*=0$. As for the bottom boundary, $z=0$, we consider that the atmosphere is coupled with the motion of the underlying water. We assume that the motion of ocean water, i.e., tsunamis, varies with horizontal scale longer than the ocean depth $D=4000$ *m* which we assume to be uniform, and weak so that the vertical displacement at the ocean surface *h*(*x*, *t*) is small compared to *D*. We used flat bottom bathymetry, because our purpose of this calculation is not to reproduce the observed waveform precisely but to demonstrate the possibility and property of tsunami resonantly excited by the internal gravity waves produced by the eruption. Then, the motion of the seawater can be described by the shallow water equations

14 $$\begin{aligned}{} & {} \frac{\partial U}{\partial t}= -g \frac{\partial h}{\partial x}-\frac{1}{\rho _w}\frac{\partial p_{atm}}{\partial x}, \end{aligned}$$

15 $$\begin{aligned}{} & {} \frac{\partial h}{\partial t}=-D\frac{\partial U}{\partial x}, \end{aligned}$$

where *U*(*x*, *t*) is the horizontal velocity of water, and $p_{atm}(x,t)$ is the atmospheric pressure at the sea surface, and $\rho _w=1000$ $\text {kg}$ $\text {m}^{-3}$ is the density of water. We assume the atmosphere and ocean couple on the sea surface as

16 $$\begin{aligned} p_{atm}(x,t)=p'(x,0,t), \end{aligned}$$

in (10) and (14) and

17 $$\begin{aligned} w^*(x,0,t)=\rho _0\frac{\partial h(x,t)}{\partial t}=-\rho _{0} D\frac{\partial U}{\partial x} \end{aligned}$$

as the bottom boundary condition of (Eq. 14).

#### Setup of numerical experiment and computational details

The computational domain covers 3200 km horizontally and 480 km vertically. The cyclic boundary condition is assumed horizontally. The basic state temperature structure is a combination of a representative profile in wintertime Japan below 100 km and a standard atmosphere above approaching exosphere temperature of 1000 K. The whole of the atmosphere is considered to be homogeneous with the standard property of air at the ground surface-atmosphere for simplicity. In the uppermost 100 km, to prevent the reflection of waves from the top boundary, the vertical velocity is damped by including the last term in (Eq. 5), where $\varepsilon$ increases linearly to 0.25 $\text {s}^{-1}$ at $z=480$ km. Vertical derivatives are evaluated using a centered finite difference scheme with the grid spacing of 250 m, whereas horizontal derivatives are calculated using a spectral method with the truncation wavenumber of 1279, which corresponds to the spatial resolution of about 800 m. Time integration is proceeded by the leapfrog scheme with the time step of 0.2 s. The model is coded using the framework of SPMODEL18. We performed another calculation with double resolution. The result is effectively indistinguishable from those presented above.

The model atmosphere, and resultingly the ocean below, is forced by localized transient heating that takes place in the earliest time interval $\tau$ of the time integration mimicking the effect of volcanic eruption expressed as

18 $$\begin{aligned} Q(x,z,t)=\frac{Q_{0}c_{p}T_{0}}{(2\tau /\pi )} \times N(x_0,\sigma _{x})\times N(z_0,\sigma _{z})\times \sin {\frac{\pi t}{\tau }}, \end{aligned}$$

where *N*(*X*, *S*) is the normal distribution centered at *X* with the standard deviation *Y*. We specify $x_0=1600$ $\text {km}$, $\sigma _x=16$ $\text {km}$, $z_0=40$ $\text {km}$,$\tau =30$ *s*, and $Q_0$ $c_p$ $T_0=10$ $\text {kg}$ $\text {m}^{-3}$ $\text {s}^{-1}$. The vague image of the eruption process behind our present choice of heating distribution in time and space is that subsurface eruption evaporates a large amount of seawater, and the resulting water vapor is carried up in the atmosphere and condensed back to water (or ice) releasing latent heat whose amount is mostly equivalent to the thermal energy of eruption. The elevation of the heating $z_0$ is tuned to roughly reproduce the ratio of the pressure amplitudes of the Lamb wave and the fastest propagating gravity wave whose phase speed is $\sim$ 250 $\text {m}/\text {s}$ observed within about 2000 $\text {km}$ from the volcano. The intensity of heating is rather arbitrary but determined so as to roughly reproduce the observed amplitude of the Lamb wave.

### Parabolic equation numerical modeling of acoustics

The parabolic equation (PE) method in wave propagation problems is used in many fields. In the case of sound wave propagation in the atmosphere, that has been used since the late 1980s 15 and is now one of the most widely accepted methods in the atmospheric acoustic field. Range-dependent propagation problems can be solved with the PE method, where factoring the wave equation into one-way equations is the solver of the PE methods. For detailed derivations and concepts, see Waxler and Assink^14^.

We used the PE model software (NCPAprop^15^) to analyze the propagation of sound waves from the Tonga volcano to Japan. Their azimuth angle of Japan is 317.5 deg measured counterclockwise from eastward, taking the origin at Tonga. Fig. 6 shows the infrasound propagation in terms of energy transmission loss, where the acoustic energy for 0.2 Hz attenuated in a range between 100 and 200 dB from Tonga to Japan (at 8000 $\text {km}$). The horizontal wind speed dataset was extracted from GEOS-5 forecasting and Horizontal Wind Model 14 (HWM14) for the upper atmosphere. Furthermore, ground to space (G2S)^16^ profiles from the Tonga eruption toward Japan were interpolated for every 0.1 degree.

## Results

**Figure 3:**
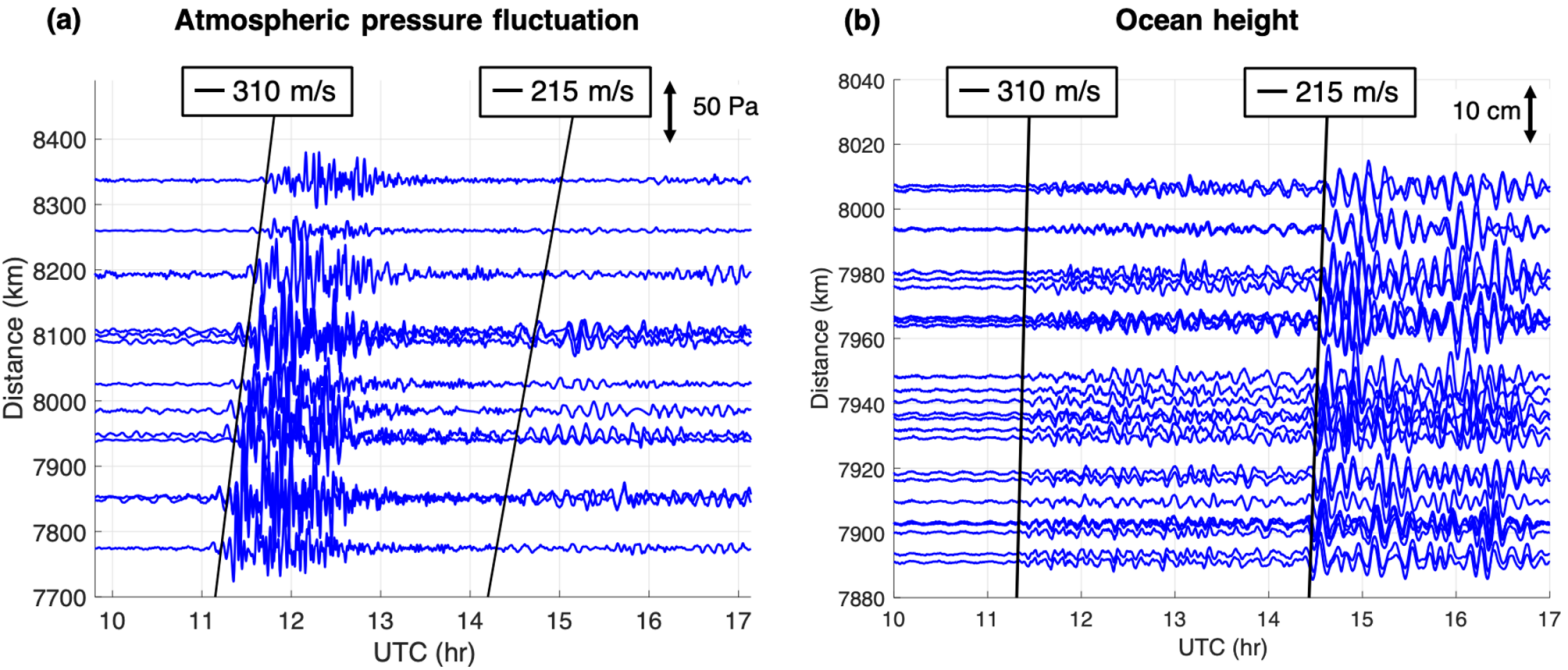
(**a**) Waveforms were obtained by the KUT infrasound network at 7700–8400 km from the Tonga volcano. A Lamb wave with a velocity of 310 m/s was observed 7 h after the eruption; the shapes of the waveforms were similar for every observation location in Japan. Several hours after the Lamb wave, long-lasting gravity waves with a velocity of 215 m/s were also observed across Japan. (**b**) The sea-level changes are calculated from DONET2 water pressure changes. DONET2 is installed near the KUT infrasound network. As in the KUT network, waves were observed with velocities of 310 m/s and 215 m/s, but the relationship with the magnitude is reversed.

The KUT sensors can detect infrasound and gravity wave signals with periods between approximately 0.2 and 1000 s, which are difficult to detect using conventional barometers. Each sensor contains a barometer, a three-component accelerometer, a thermometer, and a differential pressure gauge which is the primary infrasound sensor (microbarometer). Combining these measurements at the same site helps us understand the nature of the observed waves. Fig. 1 shows examples of the infrasound and barometer signals observed at three sites in the KUT sensor network. Usually, it is difficult to correlate waveforms at different sites because they are contaminated by local noise. However, in the case of the Tonga volcanic eruption, the similarities among the observations by the 25 KUT stations across Japan are evident even without a correlation analysis, indicating an intense coherent signal reflecting the vast scale of the eruption. The pressure waves from the Tonga eruption reached Japan, 8000 km away, approximately 7 h after the eruption. Its speed was estimated to be 310 m/s, which is interpreted as the average speed of sound for the lower atmosphere (Fig. 3a).

**Figure 4:**
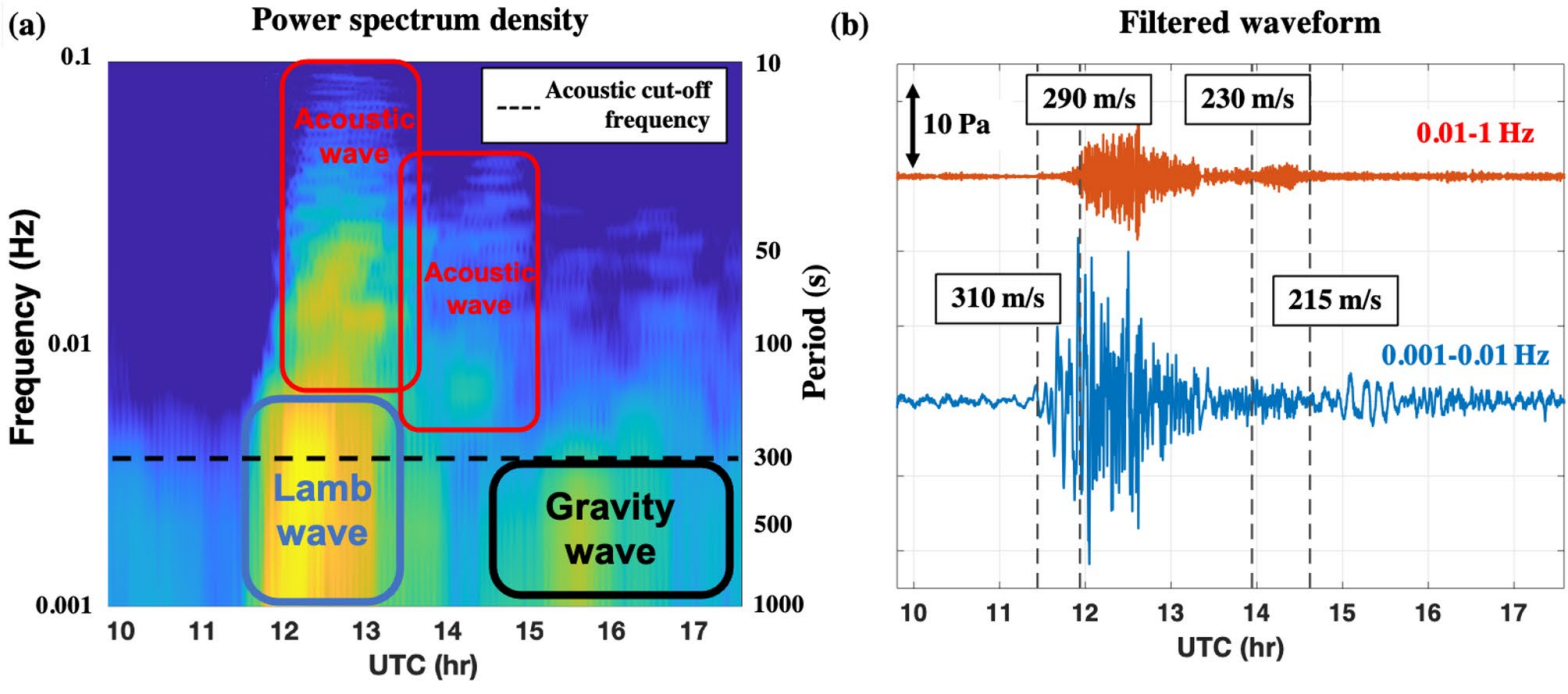
Time series of atmospheric pressure perturbations at Muroto, Kochi (N33.25, E134.18). (**a**) Power spectrum density of atmospheric pressure waves and (**b**) filtered signals in two frequency ranges. Rectangles in (**a**) indicate each wave frequency and time range. The lower-frequency Lamb wave arrives first (310 m/s), followed by the acoustic (290 and 230 m/s) and gravity waves with higher frequency. The last gravity wave, whose velocity matched that of the tsunami (200–220 m/s), caused a Proudman resonance and excited the tsunami on the Pacific Ocean.

The pressure variation continued for at least several hours afterwards. A frequency analyses of the pressure waves and the arrival time of each wave packet suggest that the wave packets are classified into four categories: one long-period Lamb wave, two short-period acoustic waves, and one long-period gravity wave (Fig. 4).

These categories have different wave velocities, which vary as functions of the distance from the sound source to the sensor. The pressure variations, which continued for several hours in Japan, were not composed of a single wave but by multiple wave packets traveling along multiple paths at different speeds constrained by the dispersion relation^17,18^ of waves under the effects of gravity and temperature inhomogeneities in the atmosphere. For example, low-frequency signals can propagate horizontally as gravity waves but cannot propagate vertically if the frequency is below the acoustic cut-off frequency (approximately 3.2 mHz at 15 $^\circ$C and at ground pressure); only high-frequency sound waves can propagate vertically. As a result, even if pressure waves emitted from the same source propagate at the speed of sound, their paths and arrival times differ depending on their frequency. The earliest arriving component seen in Fig. 4 is the Lamb wave^19^. This pressure wave is trapped within approximately 20 km above the ground surface and propagates at the average speed of sound in this region. This component has a wide frequency range that extends below the detection limit of the infrasound sensors. The barometer observation datasets show that the peak pressure change is approximately 200 Pa, and the half-waveperiod is approximately 1000 s. It is known that such low-frequency Lamb waves suffer little attenuation and can easily propagate over several thousand kilometres. Even Lamb waves that propagate from the other side of the Earth, or encircle the Earth more than once, have been observed. The observed presence of the Lamb wave component strongly implies that the eruption mainly generated Lamb waves with periods of $\geqslant$ 200 s, suggesting the large magnitude of the explosion event. The second and third arriving waves in Fig. 4 are acoustic waves with a frequency of 0.003–0.05 Hz. These waves arrived approximately 2000 and 10,000 s after the first Lamb wave, respectively. The frequency of these acoustic waves is higher than that of Lamb waves. The frequency and velocity of these acoustic waves suggest that they propagated through the middle and upper atmosphere. The second and third waves had a velocity of 290 and 230 m/s, respectively, calculated from the horizontal distance from the sound source to the sensor and the arrival time. The fourth arriving wave in Fig. 4 are gravity waves characterized by steady oscillations with a period of approximately 10 min. These waves were observed following the sound waves at 14 out of 25 sites, whose locations are indicated in Fig. 1. The lower curve in Fig. 4b, representing the lowermost-frequency component measured by the differential pressure gauge, shows the arrival of the gravity wave component more clearly. Based on the accompanying barometer measurements, the raw amplitudes of the oscillations exceeded 20 Pa at many stations. Although the waveforms differed slightly from station to station, the arrival times were earlier in the southern region and later in the north, which is consistent with the idea that a wave packet propagated as a kind of internal gravity wave at approximately 200–220 m/s away from the Tonga volcano. The atmospheric pressure variations observed in Muroto, as shown in Fig. 4, were observed throughout Japan (See Supplementary Fig. S1) because this eruption is a global event.

### Gravity-wave-induced tsunami over the pacific ocean

We found that the Lamb waves had a velocity of 310 m/s and the gravity waves had a velocity of 215 m/s (Fig. 3a). Small sea-level changes were observed at the same time as the arrival of the Lamb waves (Fig. 3b). Further, we found that large sea-level changes propagated at a similar speed to the gravity waves. This indicates that the small waves that arrived first were excited by forced oscillations from the Lamb waves, and the large waves arriving later were excited by the gravity waves. The arrival time (11:00 UTC) of the first sea-level fluctuation along the Japanese coastline was well correlated with the arrival of the wave packet containing the gravity waves (Fig. 3a, 3b). It should be noted that the gravity waved observed by the KUT sensors could be the origin of the tsunami as a result of Proudman resonance^20^. It should also be emphasized that this propagation speed is close to the typical tsunami speed in the Pacific Ocean. Both gravity and tsunami waves presumably travelled with almost the same speed of 215 m/s.

**Figure 5:**
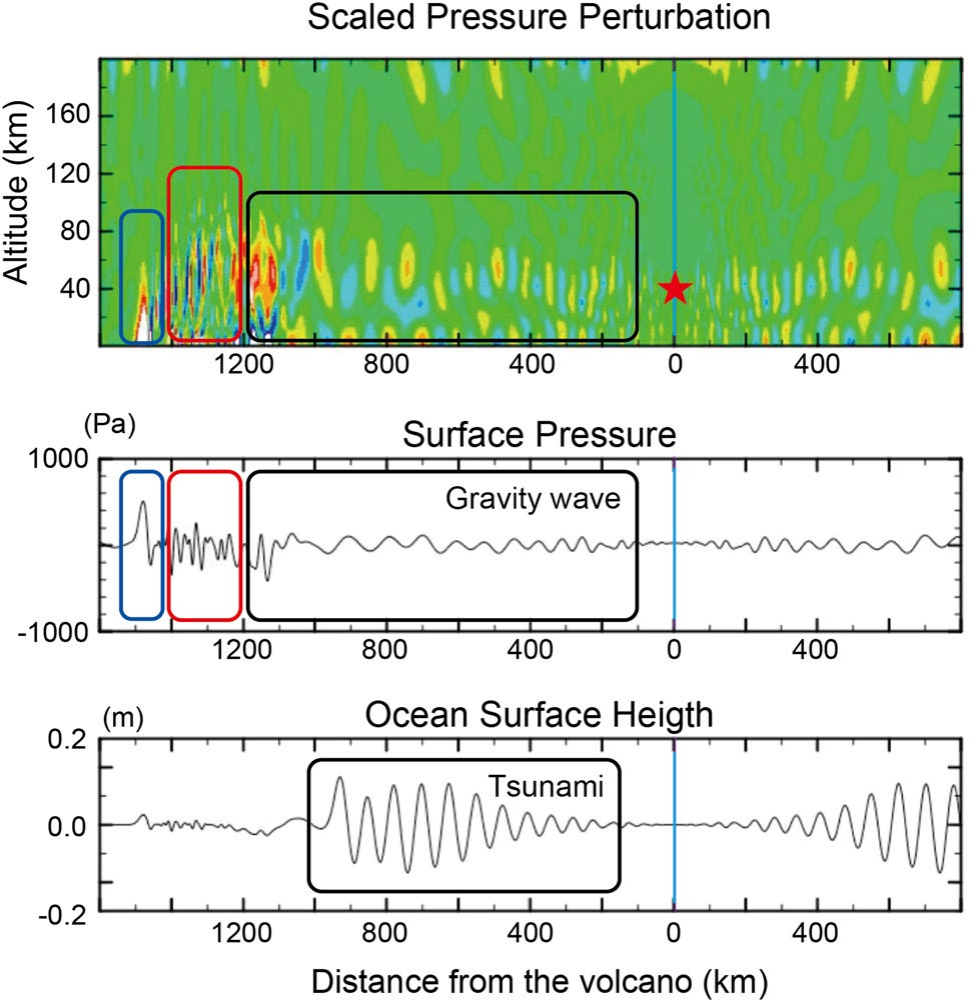
Pressure perturbations and ocean surface height simulated by a hydrodynamic model, including the coupling of the atmosphere and the tsunami. A localized heat source (indicated by a star) is initially given and excites various atmospheric waves. Lamb waves (blue rectangle) propagate at the speed of sound while trapped near the sea surface. Acoustic waves (red rectangle) immediately follow, bouncing between the mesopause (altitude of  100 km) and the sea surface. Then, gravity waves (black rectangle) follow, being trapped below the mesopause. The gravity waves propagate with a variety of velocities depending on the horizontal wavelength and vertical structure, and some of them propagate with a phase velocity of  200 m/s, resonating with tsunamis at ocean depths of 4000 m, resulting in large-amplitude tsunamis (See Supplementary Movie S1 for temporal evolution).

To investigate the interaction of gravity waves and tsunamis, we used a two-dimensional numerical model based on hydrodynamic equations describing the dynamic coupling of the compressible atmosphere and the oceanic tsunamis below it. The model shows that the explosion reaching into the stratosphere excites all the observed waves, i.e., Lamb waves that are trapped near the ground surface, acoustic gravity waves that propagate while “bouncing” between the ground and the mesopause (altitude of  90 km), and gravity waves that have a vertically modal structure between the ground surface and the mesopause and that dispersively propagate with a wide range of horizontal speeds, covering that of tsunamis. Some acoustic waves have large amplitudes in the thermosphere and possibly disturb the ionosphere^21^. Large amplitude sea level fluctuations are excited by gravity waves that are in resonance with the tsunamis despite the fact that the amplitude of the gravity waves is much smaller than that of Lamb or acoustic waves, which are off-resonance. These simulation results are consistent with the observations that the first small sea-level change arrives at the same time as the Lamb waves, and that the large sea-level change arrives at the same time as the gravity waves (Figs. 3, 5).

Notably, the gravity wave–tsunami resonance seems to occur over a relatively wide range of characteristic velocities. The time evolution (Supplementary Movie S1) reveals that tsunamis are excited by gravity waves whose phase speed is comparable to that for the tsunami ( 215 m/s), which propagate collectively as a wave packet with a group velocity that is slower than the phase speed of the tsunami. The tsunami is weak in the region close to the volcano, and gradually amplifies at a distance of several tens of kilometres from the volcano, and then remains relatively constant. This implies that the high tsunamis that struck the islands of Tonga within several kilometres require excitation mechanisms other than the gravity waves, such as pyroclastic flow and submarine landslides, whose effects will be studied in future work. It has been observed that the sea level fluctuation increased along the coast of Japan around the start time of the detected gravity wave packet. The numerical simulation implies, however, that the observed coincidence of the arrivals of gravity waves and tsunamis should be interpreted not as continuous resonance between the gravity waves and tsunamis but as their tandem propagation after resonant amplification in the region relatively close to the volcano. Still, this point remains to be verified by careful comparison between barometric and tsunami observations over the entire Pacific.

### Two packets of acoustic waves

**Figure 6:**
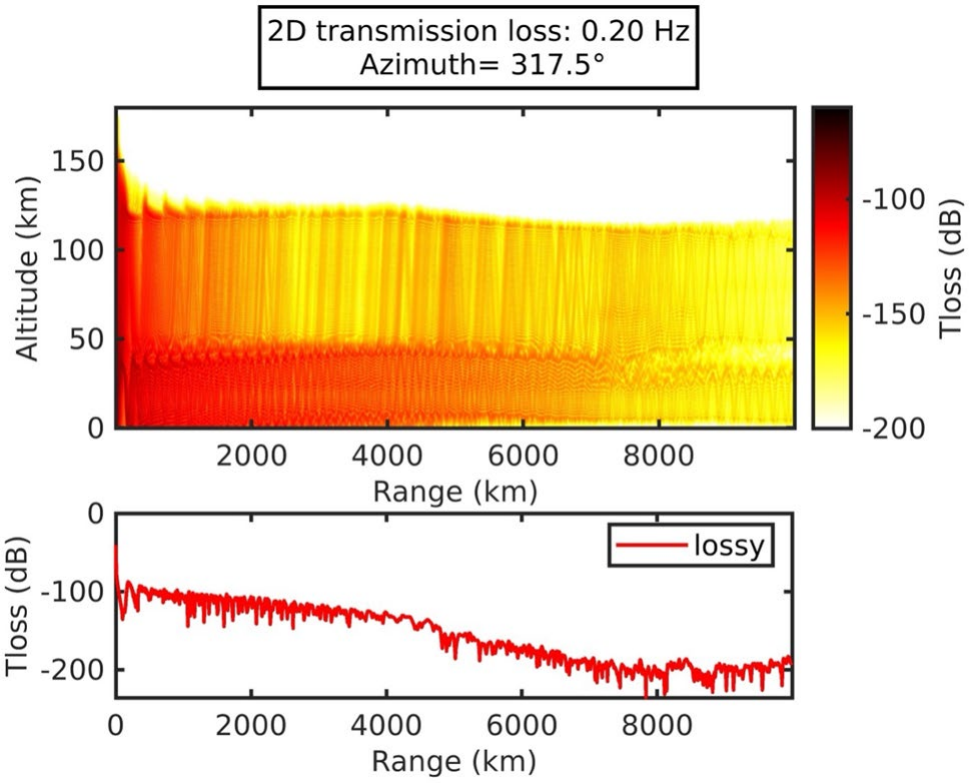
The 2D-transmission loss simulation by the parabolic range-dependent propagation method in the direction of the KUT sensors network (Azimuth = 317.5).

We have identified two kinds of ray paths for acoustic waves. One is refracted from the stratosphere; the other is refracted from the lower thermosphere. According to three-dimensional ray-tracing calculations for infrasound emitted by a volcanic eruption^18^, the velocity of some of the waves returning from the stratosphere is approximately 300 m/s, and that for waves from the lower thermosphere is approximately 238 m/s. We can explain the apparent propagation velocities based on the two-dimensional calculation of acoustic energy transmission loss in the azimuthal direction toward Japan based on a parabolic equation as shown in Fig. 6. Moreover, the sound waves returning from the lower thermosphere have a lower frequency than those from the stratosphere. In this calculation, the third wave has a lower thermospheric path and a longer propagation distance. However acoustic propagation still has some mysterious effect which will be investigated in the future work.

### Estimation of Lamb wave energy

The energy flux (W/$\text {m}^2$) of sound waves is expressed as

19 $$\begin{aligned} F=\frac{p^2}{\rho c_s} \end{aligned}$$

where *p* is the sound pressure, $\rho$ is the atmospheric density, $c_s$ is the sound speed. In the following, we assume an isothermal atmosphere. Since Lamb the wave is of the order of scale height^22^ $\gamma H$, where $\gamma$ is the specific heat ratio of atmosphere taken as $\sim$ 1.4 and H is the pressure scale height of $\sim$ 8 km, the sound pressure at an altitude of z is approximated by

20 $$\begin{aligned} p(z)\sim p(0)\exp -(\frac{z}{\gamma H}) \end{aligned}$$

Furthermore, the density$\rho$ is also approximated by

21 $$\begin{aligned} p(z)\sim \rho _(0)\exp -(\frac{z}{H}) \end{aligned}$$

Substituting (Eq. 20) and (Eq. 21) into (Eq. 19), we obtain the energy flux at an altitude of z as follows:

22 $$\begin{aligned} F(z)=\frac{p^{2}(0)\exp ^{2}(-\frac{z}{\gamma H})}{\rho (0)\exp (-\frac{z}{H})c_s}. \end{aligned}$$

Assuming that the Lamb wave is isotopically propagated as a cylindrical wave, the total energy of the Lamb wave $E_{tot}$ is given by

23 $$\begin{aligned}{} & {} E_{tot}=\int _0^\infty {2\pi r \times \frac{\frac{p^{2}(0)}{\rho (0)c_s}\exp ^2 (-\frac{z}{\gamma H})}{\exp (-\frac{z}{H})}\,dz}. \end{aligned}$$

24 $$\begin{aligned}{} & {} E_{tot}=2\pi r \times \frac{p^{2}(0)}{\rho (0)c_s} (\frac{\gamma }{2-\gamma })H \times \frac{T}{2}, \end{aligned}$$

where *r* is the distance from the sound source, and *T* is the duration time. Finally, we get the yield estimation (Table 1) by substituting the observed value based on the positive peak pressure amplitude of the Lamb wave in Eq. (24).

**Table 1:** The estimated yield from barometric observations within 8000 km from Tonga.

Site	Yield (Megaton)	Distance (km)	Pressure (Pa)	Duration (s)
FUNA	63.9	1452	540	1650
AFI	42.3	829	590	1560
MSVF	63.1	756	660	2040
SNZO	53.9	2484	380	1660
GUMO	47.4	5759	240	1700
TARA	46.1	2737	350	1560
MAJO	29.9	7980	180	1630

## Discussion

We determined the propagation characteristics of waves excited by the global-scale volcanic eruption that occurred in Tonga in January 2022. Four wave packets were observed to be similar among the 25 observation sites in Japan. These were categorized as Lamb waves, two paths of acoustic waves, and atmospheric gravity waves. The Lamb waves and our simple equation provide the yield and a lower estimation of the explosive energy of the Tonga eruption. To reach Japan from the site of the volcano, the acoustic waves are inferred to have bounced several times on the sea surface; that is, the infrasound travelled back and forth between the atmosphere and the ocean surface multiple times. This significant eruption allowed us to observe global vertical energy exchanges in the atmosphere. The observed Lamb and acoustic waves are of great interest to the geoscience community, but the last gravity wave pertains to a more prominent theme, as it excited an unexpected tsunami in the Pacific Ocean. The velocity of the gravity waves was in good agreement with the velocity of tsunamis, resulting in the coupling of the gravity waves with the tsunamis, which consequently generated a second wave of unpredicted tsunamis.

## Conclusion

Tonga eruption generated several types of atmospheric pressure. KUT sensors network recorded these signals over 8000 km in Japan. The infrasound-detected signals can be classified as gravity wave, Lamb wave, and acoustics. This study proved these observations using different models based on the nature and the characterization of each packet. In addition, the subsequent tsunami, which followed the arrival of Lamb wave within a few hours at the Japanese coastline, was confirmed by the gravity wave modeling. The arrival of the high-frequency signals (acoustics) was proved by PE modeling toward Japan. Moreover, the yield energy was estimated from the observed Lamb wave over the Pacific Ocean with a mean of 49.3 Megaton. The tsunami was observed even in the Caribbean Sea, a body of water separated from the origin of the eruption by the continent of North America. In other words, tsunamis could be generated by gravity waves from anywhere on Earth. Thus, gravity waves should be considered important not only for geoscience but also for disaster mitigation.

## Supplementary Information

Supplementary Information 1.

Supplementary Information 2.

## Data Availability

The data that support the findings of this study are openly available from the ’Kochi University of Technology Infrasound Observation Network System’ at https://geosci.mydns.jp/infrasound/graph.php (last visit: 20/11/2022). The source code for the numerical model used for the atmosphere-tsunami coupling simulation can be obtained from K.N. upon reasonable request. The GEOS data used in this study/project have been provided by the Global Modeling and Assimilation Office (GMAO) at NASA Goddard Space Flight Center through the online data portal in the NASA Center for Climate Simulation.
