# Supplementary material for: Observation and simulation of atmospheric gravity waves exciting subsequent tsunami along the coastline of Japan after Tonga explosion event
Source: Sci Rep. 2022 Dec 26;12:22354. doi: 10.1038/s41598-022-25854-3 (PMC9792542; doi:10.1038/s41598-022-25854-3)
Supplement: Supplementary file 1 — Supplementary Information 1. [file 41598_2022_25854_MOESM1_ESM.pdf]

Supplementary Materials
Observation and Simulation of Atmospheric Gravity Waves Exciting Subsequent Tsunami along the Coastline of Japan after Tonga Explosion Event

1- Fig. 1 shows the power spectrum density from several KUT infrasound sites all over Japan.

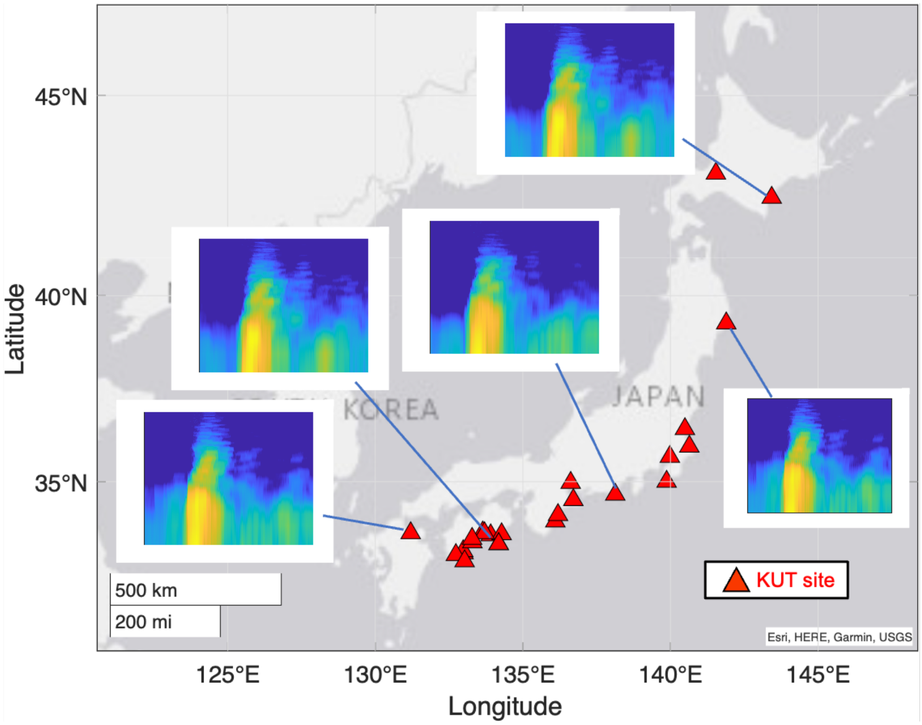

Figure 1: The power spectrum density of five KUT infrasound sites which are located in different areas of Japan. The map is created with Matlab R2022B (www.mathworks.com)

2- The supplementary movie shows the temporal evolution of the atmospheric waves and tsunamis simulated in the hydrodynamic model:
(top) the scaled pressure distribution in the atmosphere with warm (cold) colorings for positive (negative) deviation, (middle) the horizontal distribution of surface pressure, and, (bottom) the horizontal distribution of ocean surface height. Variable at every 60 seconds are shown.
